# Comparative Study on the Antioxidant Activity of *Monascus* Yellow Pigments From Two Different Types of *Hongqu*—Functional Qu and Coloring Qu

**DOI:** 10.3389/fmicb.2021.715295

**Published:** 2021-08-02

**Authors:** Li Wu, Kangxi Zhou, Feng Chen, Guimei Chen, Ying Yu, Xucong Lv, Wen Zhang, Pingfan Rao, Li Ni

**Affiliations:** ^1^College of Chemistry, Fuzhou University, Fuzhou, China; ^2^Institute of Food Science and Technology, College of Biological Science and Engineering, Fuzhou University, Fuzhou, China; ^3^Research Institute of Agri-Engineering and Technology, Fujian Academy of Agricultural Sciences, Fuzhou, China

**Keywords:** *Hongqu*, *Monascus* azaphilone pigments, *Monascus* yellow pigment, antioxidant activity, cellular antioxidation

## Abstract

This study is the first to investigate the difference in the composition of *Monascus* azaphilone pigments (MonAzPs) between functional Qu (FQ) and coloring Qu (CQ) and analyze their relationships with antioxidant activity. The composition of key active components and antioxidant activity of the ethanol extracts of FQ and CQ were analyzed by Uv-vis, HPLC, and chemical antioxidant tests. The composition of MonAzPs of the ethanol extracts was further analyzed by HPLC-MS. Seven *Monascus* yellow pigments (MYPs) with high abundance were successfully purified for the antioxidation evaluation *in vitro* and in the cell. Correlation analysis between the metabolites and the antioxidant activity of *Hongqu* indicated that MonAzPs might play an essential role in the antioxidant activity (*r* > 0.80). By contrast, the monacolin K (MK), polysaccharide, ergosterol, and γ-aminobutyric acid (GABA) were not significantly correlated with the antioxidant activity. Orthogonal partial least squares discriminant analysis (OPLS-DA) based on the composition of MonAzPs revealed that the abundance of MYPs is significantly different between FQ and CQ (*P* < 0.05 and *VIP* > 1.0). Seven MYPs (monasfluore A, monaphilone B, monascuspilion, monascin, monaphilone A, ankaflavin, and new yellow pigment) with high abundance were successfully purified for the antioxidation evaluation. Chemical antioxidant tests revealed that the antioxidant activities of monaphilone A, ankaflavin, and new yellow pigment only from CQ were significantly more potent than monasfluore A and monascuspilion only separated from FQ. The cellular antioxidant assay (CAA) showed that the new yellow pigment had the best antioxidant activity (quercetin equivalent 7.23 μM), followed by monasfluore A and monaphilone B, all of which were significantly better than monascin and ankaflavin, the two most frequently reported MYPs. Research on the structure–activity relationship demonstrated that alterations of the hydroxyl that occurred on C-3′ or C-11 obviously affected the antioxidant activities of MYPs. Our findings provide evidence that MYPs may be the key active components for CQ to have a more potent antioxidant capacity than FQ. The alterations of the hydroxyl that occurred on C-3′ or C-11 obviously affected the antioxidant activities of MYPs.

## Graphical Abstract

Monascus yellow pigments (MYPs) in functional Qu (FQ) have a higher antioxidant potential than those in coloring Qu (CQ). The difference in antioxidant activities of MYPs is due to the critical hydroxyl group on C-3′ or C-11.

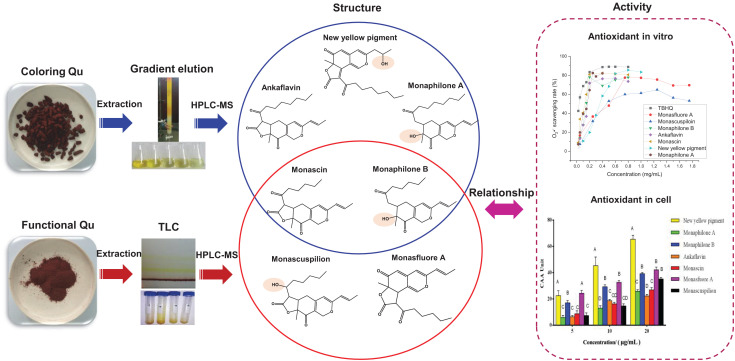

## Introduction

*Hongqu* (rice fermented by *Monascus*) is used as medicine and food for thousands of years in Asia (Huang Z. et al., 2019). According to the different *Monascus* strains (*Monascus pilosus* for FQ and *Monascus purpureus* for CQ) and fermentation processes (Supplementary Figure 1), *Hongqu* is mainly divided into functional Qu (FQ) and coloring Qu (CQ). FQ is rich in Monacolin K (MK, over 4.0 mg/g)(National Development and Reform Commission of the People’s Republic of China, 2007), and CQ is rich in *Monascus* Azaphilone pigments (MonAzPs, over 1000 U/g)(National Health and Family Planning Commission of the People’s Republic of China, 2015). FQ and CQ are fermented with different *Monascus* strains with excellent pigment and MK production ability, respectively. Besides, CQ is generally fermented in a large pond for 5–7 days, and FQ is generally fermented in an Erlenmeyer flask (500 or 1,000 ml) for 35–42 days. Because of the differences in the fermentation strains and fermentation processes, the fermented products (FQ and CQ) have different metabolite compositions and functional characteristics. The ancient medical work “*Compendium of Materia Medica*” recorded that *Hongqu* has the beneficial effects of promoting blood circulation and removing blood stasis. Modern medical researches verified and explored multiple pharmacological activities of *Hongqu* and its metabolites, including hypolipidemia (Wang and Pan, 2003; Lee and Pan, 2012; Lee C. L. et al., 2013), hypoglycemia (Shi et al., 2012; Zhou et al., 2019), and anti-aging (Chen et al., 2015). Certain correlations between those pharmacological activities and antioxidation had been found with the in-depth study. The mechanism may be related to that special molecular structure transferring hydrogen to free radicals or accepting free radical electrons (Zhang et al., 2020), which can scavenge excessive free radicals and maintain redox balance. The study of antioxidant activity has been the basis for other activities. The antioxidant activity of *Hongqu* may be related to the key active components of *Monascus*, including MonAzPs, MK, γ-aminobutyric acid (GABA), ergosterol, and polysaccharides. However, the effects or contribution of the metabolites in *Hongqu* on the antioxidant activity needs to be further evaluated.

Among the metabolites in *Hongqu*, MonAzPs have excellent antioxidant capacity (Qu et al., 2008; Chen et al., 2017), of which MYPs have the most potent antioxidant capacity (Zhang et al., 2020). A previous study found that ANKASCIN 568 plus (monascin and ankaflavin as the main functional components) may increase the antioxidant enzyme activities and inhibit the oxidation reaction induced by amyloid Aβ, thereby significantly improving the memory and learning ability of Alzheimer’s (Chen et al., 2015). Besides, monascin may inhibit the oxidative stress caused by the phosphorylation of peroxisome proliferator-activated receptor-γ (PPARγ) by attenuating the activation of protein kinase C (PKC), thereby repairing pancreatic damage (Hsu W. H. et al., 2013). The antioxidant activity has been further confirmed to be closely related to other pharmacological activities (Lee C. L. et al., 2013). The relationship between MonAzPs (especially MYPs) and antioxidant activity needs to be further explored to promote the development and utilization of MYPs antioxidant resources.

In this study, the difference of key active components in *Hongqu* (FQ and CQ) and their relationship with antioxidant activity were compared. Besides, the composition of MonAzPs and the *in vitro* antioxidant activity (chemical antioxidant tests and cellular antioxidant assay) of MYPs isolated from two types of *Hongqu* were further investigated. Finally, the relationship between the antioxidant activities and the structure of MYPs was also analyzed, which laid the foundation for the research on the antioxidant activity of MYPs and also provided a reference for studying the antioxidative related effects of MYPs.

## Materials and Methods

### Material

**FIGURE 1:**
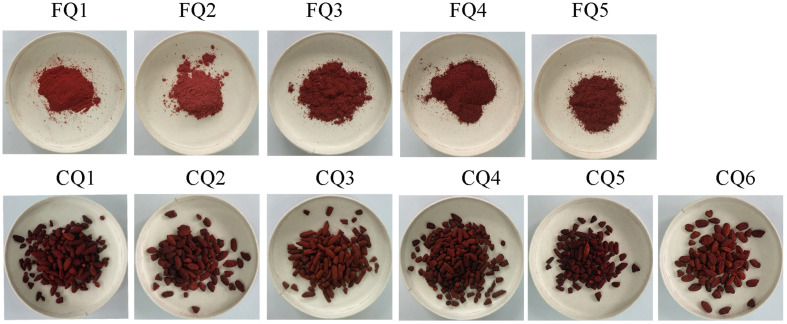
Appearance of *Hongqu*. FQ1–FQ5 were functional Qu (FQ). CQ1–CQ6 were coloring Qu (CQ).

*Hongqu* samples were mainly collected from Ningde City and Fuzhou City in Fujian Province, and Jinhua City and Jiangshan City in Zhejiang Province, including five samples with MK over 4.0 mg/g (also named functional Qu, FQ1–5) and six samples with pigment over 1,000 U/g (also named coloring Qu, CQ1–6) (Table 1). The appearance of *Hongqu* showed that the shape of FQ was powder and CQ was rice grains, and the color of CQ was redder and darker than FQ (Figure 1).

**TABLE 1:** The source and region of *Hongqu*.

Hongqu	Source	Region	Remarks
FQ1	Zhejiang Sanhe Biotech Co., Ltd.	Jiangshan City, Zhejiang Province	MK 3.0%
FQ2	Zhejiang Sanhe Biotech Co., Ltd.	Jiangshan City, Zhejiang Province	MK 1.5%
FQ3	Fuzhou University	Jinhua City, Zhejiang Province	MK 0.5%
FQ4	Fujian Outlet Biotech Co., Ltd.	Fuzhou City, Fujian Province	MK0.5%
FQ5	Fujian Outlet Biotech Co., Ltd.	Fuzhou City, Fujian Province	MK 1.0%
CQ1	Fujian Pinghuhong Bio-tech Co., Ltd.	Ningde City, Fujian Province	Color value 1,000 U/g
CQ2	Fujian Chengjiu *Hongqu* Co., Ltd.	Ningde City, Fujian Province	Color value 1,300 U/g
CQ3	Fujian Pinghuhong Bio-tech Co., Ltd.	Ningde City, Fujian Province	Color value 2,000 U/g
CQ4	Fujian Pinghuhong Bio-tech Co., Ltd.	Ningde City, Fujian Province	Color value 4,000 U/g
CQ5	Fujian Chengjiu *Hongqu* Co., Ltd.	Ningde City, Fujian Province	Color value 500 U/g
CQ6	Fujian Chengjiu *Hongqu* Co., Ltd.	Ningde City, Fujian Province	Color value 3,500 U/g

### Analysis of the Composition of Key Active Components

#### Determination of the Total Color Value

The pigments quantification was done with a UV-vis spectrophotometer (CLARIOstar, BMG LABTECH GmbH, Ortenberg, Germany). The total color value was assessed by measuring the absorbance of 75% ethanol extract of *Hongqu* at 505 nm. The result was expressed as the absorbance unit per gram at a specific wavelength (Srianta et al., 2016).

#### Determination of MK by HPLC

Determination of MK by HPLC was in accordance to the method in QB/T 2847-2007 and reference (Huang C. F. et al., 2019) with minor modification. Before chromatographic analysis, the supernatant was filtered through a 0.22 μm filter. The supernatant was measured by Waters e2695 with a chromatographic column (Zorbax SB-C18 column, 5 μm × 250 mm × 4.6 mm) and a UV detector at 238 nm. The mobile phase was composed of methanol:water:phosphoric acid in a ratio of 385:115:0.14 (v/v), with an accompanying flow rate of 1.0 ml/min.

#### Determination of γ-Aminobutyric Acid by HPLC

γ-Aminobutyric acid was extracted and analyzed according to the method in QB/T 4587-2013 (Ministry of Industry and Information Technology of the People’s Republic of China, 2013). The content of GABA was determined by HPLC equipped with a reversed-phase column (Novapack C18, 300 mm × 3.9 mm × 4 μm, Waters, Milford, MA, United States). Eluted GABA was detected at 280 nm and expressed as mg/100 g dry *Hongqu* (Garzón et al., 2020).

#### Determination of Ergosterol by HPLC

The content of ergosterol was determined by HPLC equipped with an ODS18 column (250 mm × 4.6 mm × 5 μm, Waters, Milford, MA, United States) at 280 nm. Ergosterol in *Hongqu* was identified by a combination of the retention time in HPLC chromatograms with standards (Guan et al., 2016).

#### Determination of Polysaccharides by HPLC

The preparation of *Hongqu* polysaccharides was under the method in the literature (Xie et al., 2012). The polysaccharide was detected by the phenol-sulfuric acid method (Dubois et al., 1956).

#### Composition of MonAzPs

Determination of pigment compounds was in accordance to the method (Liang et al., 2019) with some modifications. The analysis was conducted by Agilent 2000 HPLC system (Agilent Technologies, Santa Clara, CA, United States) comprised of a binary pump, an autosampler, and a thermostatically controlled column compartment. Pigments were separated on a 250 × 4.6 mm ODS18 column (5 μm, Waters, Milford, MA, United States) with a linear gradient of the mobile phase of solvent A (water, containing 0.1% formic acid, v/v) and solvent B (acetonitrile), a flow rate of 1.0 ml/min, oven temperature of 35°C, and run time for 55 min. Mass spectrometry was operated with a 6520 QTOF-MS (Agilent Technologies, Santa Clara, CA, United States) equipped with an ESI source. The parameters of the ESI source were set as follows: capillary voltage, 3,500 V; fragmentor voltage, 135 V; drying gas (N2) temperature, 350°C; drying gas (N_2_) flow rate, 10.0 L/min; nebulizer gas pressure, 30 psig; OCT RF V, 750 V; skimmer voltage, 65 V. Each sample was analyzed in positive modes with a mass scan range of m/z 50–1,200 Da, and collision energy (CE) was set as 15 and 30 eV. Data acquisition and analysis were operated by Agilent LC/MS Qualitative Analysis Software (version B.04.00).

### Antioxidant Activity of Hongqu and MYPs *in vitro*

#### DPPH Free Radical Scavenging Experiment

The DPPH radical scavenging capacity was determined according to the method described by reference (Brand Williams et al., 1995) with minor modification. Each sample with a fixed volume of 1.0 ml was mixed with 1.0 ml DPPH solution (0.2 mM in anhydrous ethanol) stored in the dark at room temperature for 30 min. Then, its absorbance A_i_ was measured at 517 nm using a microplate reader (CLARIOstar, BMG LABTECH GmbH, Ortenberg, Germany). At the same time, the absorbance A_*c*_ of the mixture of DPPH solution and an equal volume of anhydrous ethanol and the absorbance A_*j*_ of the mixture of the sample solution and the equal volume of anhydrous ethanol were determined at 517 nm. The DPPH radical scavenging activity was expressed as the percentage of inhibition of DPPH according to the following formula. Inhibition rate of DPPH = [1- (A_i_-A_*j*_)/A_*c*_] × 100%.

#### ABTS Free Radical Scavenging Experiment

ABTS radical scavenging ability was determined according to the method with some modifications (Sridhar and Charles, 2019). The ABTS⋅^+^ working solution was produced by reacting the ABTS⋅^+^ stock solution (7.4 mM ABTS) with the K_2_S_2_O_8_ stock solution (2.6 mM K_2_S_2_O_8_) in equal quantities and allowed them to react for 12–16 h at room temperature in the dark and diluting with anhydrous ethanol to make its absorbance 0.70 ± 0.02 at 734 nm. ABTS⋅^+^ working solution (1.0 ml) was mixed with 0.25 ml of the ethanol extract of *Hongqu* or standard (dibutyl hydroxytoluene, BHT) at different concentrations (0.1–10 μg/ml). The mixture was incubated at room temperature for exactly 10 min in the dark and determined the absorbance at 734 nm (A). The control (A_0_) was prepared by mixing 1.0 ml of ABTS⋅^+^ solution with 0.25 ml of anhydrous ethanol. The percentage results of scavenging activity were calculated as inhibition rate using the following equation. Inhibition rate of ABTS⋅^+^ = [(A_0_ − A)/A_0_] × 100%.

#### Ferrous-Reducing Power

Ferrous-reducing power was determined as described in the literature (Eda et al., 2016) with some modifications. Sample solution (0.25 ml), phosphate buffer (0.25 ml) (pH 6.6, 0.2 mol/L), and potassium ferricyanide (0.25 ml) (10 g/L) were placed in a 1.5 ml centrifuge tube. After incubation at 50°C for 20 min and cooling to room temperature, 0.25 ml of 10% (m/v) trichloroacetic acid (TCA) solution was added and centrifuged at 4°C and 10,000 r/min for 15 min. Supernatant (0.5 ml), distilled water (0.5 ml), and 0.1% (m/v) ferric chloride solution (0.1 ml) were added and reacted for 10 min. The absorption was measured at 700 nm.

#### Superoxide Anion Free Radical Scavenging Experiment

The superoxide anion was produced by the AP-TEMED system and reacted with hydroxylamine hydrochloride to form NO_2_^–^. Then, NO_2_^–^ was reacted with paminobenzenesulfonic acid and α-naphthylamine to form a red azo compound with a characteristic absorption peak at 530 nm (Takei et al., 2017). The scavenging ability of oxygen anions was negatively correlated with the absorbance at 530 nm. O^2–^ radical-scavenging activity was measured by a non-enzymatic method with a test kit produced by Beijing Solarbio Science and Technology Co., Ltd.

#### Determination of the Inhibition Rate of Yolk Lipoprotein (AOA%)

The inhibition rate of yolk lipoprotein (AOA%) was determined as described in our previous report (Wu et al., 2016). Phosphoric acid buffer (3.0 ml) (0.1 mol/L), 1:25 yolk suspension (0.4 ml), FeSO_4_ solution (0.2 ml) (25 mmol/L), and sample solution (0.2 ml) were placed in 10 ml tubes, respectively. The control tube (A_0_) was placed with 0.2 ml of phosphoric acid buffer instead of the sample solution (A). After incubation at 37°C for 12 h, 1.0 ml of 20% trichloroacetic acid was added, centrifuging for 10 min at 3,500 r/min. Supernatant (4.0 ml) was reacted with 2.0 ml of 0.8% thiobarbituric acid solution for 15 min at 100°C. The absorbance was determined at 532 nm and calculated by the following formula, Yolk lipoprotein inhibition rate (AOA%) = [(A_0_ − A)/A_0_] × 100.

### Determination of the Cellular Antioxidant Activity of MYPs

Cellular antioxidant measurements were determined following the method (Wolfe and Liu, 2007) with modifications. Caco-2 cells in the logarithmic growth phase were inoculated into 96 well cell culture plates at the density of 6 × 10^4^ cells/well. The growth medium was removed, and the cells were washed with HBS to remove any non-adherent and dead cells. Next, 50 μl DCFH-DA working solution (25 μM) was added to each well, followed by 50 μl of the sample solution (in triplicate wells). For positive control, 50 μl of quercetin (the final dilution concentration was 2, 4, 6, 8, and 10 μM) were applied in triplicate wells, while the blank group was treated with DCFH-DA without ABAP. Once the DCFH-DA and antioxidant treatments were added, the cells were placed in the incubator for 1.0 h at 37°C. After this period, the cells were washed with HBS, and DCFH-DA was removed. Then, each well was added with 100 μl ABAP (600 μM). The cells were immediately placed in a FlexStation3 multifunctional calcium flow detection workstation (Molecular devices, United States), where real-time fluorescence was read initially and every 5 min, then after for 1.0 h. Fluorescence was measured at an excitation wavelength of 485 nm and an emission wavelength of 538 nm (Zhou et al., 2020).

$$\begin{array}{l} \mathrm{The}\mathrm{formula}\mathrm{was}\mathrm{as}\mathrm{follows}:\mathrm{CAA}\mathrm{unit}=\%\mathrm{reduction}\\ =100-\left(\int \mathrm{SA}-\int \mathrm{CA}\right)\times 100 \end{array}$$

where ∫SA was the integrated area under the sample fluorescence vs. time curve and ∫CA was the integrated area from the control curve.

### Isolation of MYPs From Crude Extracts of FQ and CQ

Coloring Qu sample was prepared according to 0.1 g/ml CQ ethanol extracts: 200–300 mesh silica gel = 2:1 (v/m), mixed and dried at 50°C for 48 h. Then, CQ sample was located on a silica gel column and eluent with the solvent of n-hexane/ethyl acetate (5:1, v/v) to yield yellow pigments. The similar fractions were combined into five main fractions according to OD 410 nm and 300–600 nm scan, and the solvent was removed under reduced pressure. The ethyl acetate phase of the FQ sample was prepared and repeated three times according to the rate of 0.1 g/ml FQ ethanol extracts:ethyl acetate:double distilled water = 1:2:3 in a 500 ml separating funnel and isolated by TLC (developing solvent was n-hexane:ethyl acetate:petroleum ether = 30:17:8), The band with the same R_f_ (drew under UV 365 nm light) was collected. These resultant fractions were further analyzed by HPLC, and then fractions with the same retention time were combined, and the solvent was removed under reduced pressure. The obtained seven yellow pigments, namely, monaphilone B, monascin, monascuspilion, monasfluore A, new yellow pigment, monaphilone A, and ankaflavin, were dissolved in ethanol for HPLC-QTOF-MS analysis and antioxidative activity determination. The HPLC diagram, mass spectrum, and structure diagram of MYPs were shown in Supplementary Figures 2–8.

### Statistical Analysis

The measurement data were expressed as mean value ± standard deviation (mean ± SD). Single-factor variance analysis and spearman’s correlations coefficient were calculated by using SPSS 22.0 (SPSS Inc., United States). Bioplot diagram, permutation test diagram of orthogonal partial least squares discriminant analysis (OPLS-DA), VIP diagram, and HCA diagram were drawn using the SIMCA-14.1 (UMETRICS, Sweden) based on the relative abundance of MonAzPs determined with HPLC-MS.

## Results and Discussion

### Analysis of the Composition of Key Active Components of FQ and CQ

#### The Total Color Value

**FIGURE 2:**
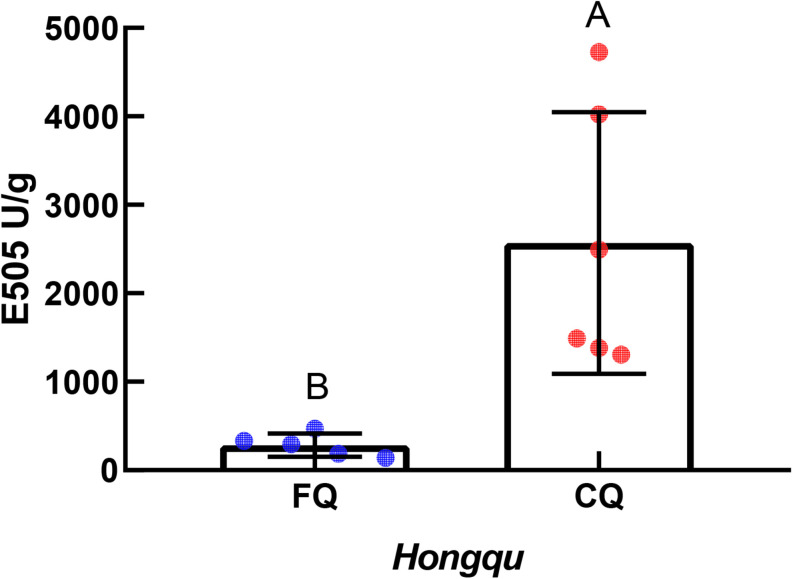
Units of absorbance at 505 nm (E_5__05_) of functional Qu (FQ) and coloring Qu (CQ), respectively.

According to Figure 2, the units of absorbance at 505 nm (E_505_) of CQ, indicating the total color value, were extremely significantly higher than those of FQ. The average value of E_505_ of CQ was approximately 2,569.60 U/g, while that of FQ was only 283.24 U/g. The E_505_ of CQ was approximately 9.0 times compared with that of FQ. For example, Huang et al. (2017) reported a higher antioxidative potential in a fermented mixture of Radix Puerariae and rice than in that fermented by only Radix Puerariae and rice due to the higher levels of pigments. The total color value of red yeast rice (RYR) and fermented Radix Puerariae and rice (FPR) was 1,300 ± 25 units/g and 6,164 ± 799 units/g, respectively. FPR increased the color value by three times compared with RYR. The result indicated that the total color value between CQ and FQ was different, so the differences in *Monascus* pigments composition needed to be further analyzed.

#### MK

Monacolin K is one of the well-documented metabolites of *Monascus* and is recognized as a cholesterol-lowering regent because of its competitive inhibitory effect on 3-hydroxy-3-methylglutaryl coenzyme A (HMG-CoA) reductase. The total amount of MK is composed of the lactone form and the acid form of MK. The content of MK in *Hongqu* was shown in Table 2. The total contents of MK of FQ 1–FQ 5 were 33.73, 20.27, 6.98, 9.02, and 10.66 mg/g, respectively. So, FQ 1–FQ 5 met the requirements of the light industry standard of the People’s Republic of China that the MK of FQ should be over 4.0 mg/g. The ratios of the active open-hydroxyl acid form and the prodrug lactone form of MK of FQ 1- FQ 5 were 13.64, 12.88, 26.93, 18.18, and 27.02%, respectively. The MK of CQ 4 was the highest among all CQ samples, reaching 0.26 mg/g (less than the requirements of FQ). Gum et al. (2017) reported higher antioxidant activity in *Hongqu* fermented *Bacillus subtilis* than in *Hongqu* due to the alteration in the physicochemical property of MK.

**TABLE 2:** The content of MK in *Hongqu* (mg/g).

Hongqu	Lactone forms of MK	Acid forms of MK	The total content of MK
CQ4	–	0.26 ± 0.01	0.26 ± 0.01
FQ1	29.13 ± 0.02	4.60 ± 0.01	33.73 ± 0.00
FQ2	17.66 ± 0.08	2.61 ± 0.02	20.27 ± 0.11
FQ3	5.11 ± 0.01	1.88 ± 0.02	6.98 ± 0.03
FQ4	7.38 ± 0.04	1.64 ± 0.03	9.02 ± 0.07
FQ5	7.78 ± 0.04	2.88 ± 0.01	10.66 ± 0.05

*Use “–” to indicate that it cannot be detected.*

#### GABA, Polysaccharides, and Ergosterol

**FIGURE 3:**
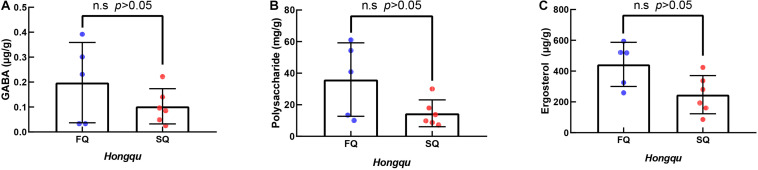
The γ-aminobutyric acid (GABA), polysaccharides, and ergosterol of functional Qu (FQ) and coloring Qu (CQ), respectively. **(A)** The γ-aminobutyric acid. **(B)** Polysaccharides. **(C)** Ergosterol.

The contents of GABA, polysaccharide, and ergosterol of CQ were 0.103, 14.58, and 246.92 mg/g, while those of FQ were 0.198, 35.97, and 443.65 mg/g, representing increases of 92.23, 146.70, and 79.67%, respectively. The contents of GABA, polysaccharide, and ergosterol of FQ were higher than those of CQ. However, there was no significant difference between them (Figure 3). Lee et al. (2016) reported that amino acids contributed to antioxidant activity in wheat and rice gochujang. Lu et al. (2015) reported that *Huangjiu* had the highest antioxidant capacity among the three traditional fermented wines (*Baijiu*, *Huangjiu*, grape wine) because the amino acid content was 2,923 μg/ml, and GABA was 10 μg/ml. Numerous studies reported that polysaccharides from different sources and modified products had certain antioxidant activities (Mirzadeh et al., 2020). Liang et al. (2019) reported that the ergosterol in RYR could significantly reduce the levels of total cholesterol, triglycerides, and low-density lipoprotein cholesterol in C57BL/6J mice fed a high-fat diet. Therefore, GABA, polysaccharides, and ergosterol might be potential contributors to antioxidant activity.

### *In vitro* Antioxidant Activity of Ethanol Extracts of FQ and CQ

**FIGURE 4:**
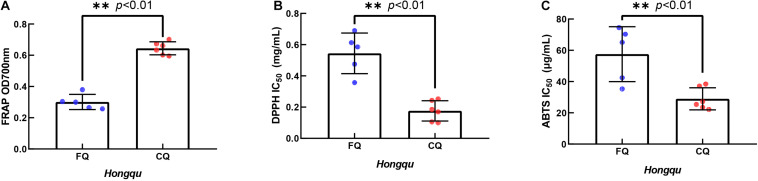
*In vitro* antioxidant activity of extracts of functional Qu (FQ) and coloring Qu (CQ), respectively. **(A)** The ferric ion reducing antioxidant power of FQ and CQ. **(B)** The DPPH radical scavenging activity of FQ and CQ. **(C)** The ABTS radical scavenging activity of FQ and CQ.

The ferric ion reducing antioxidant power (FRAP) of 1.0 mg/ml ethanol extracts of FQ and CQ was measured (Figure 4A). The average FRAP of CQ was 0.645, which was significantly better than that of FQ (0.301). When the FRAP of 1.0 mg/ml vitamin C was 0.918, the FRAP of CQ 4 was the strongest among all samples, reaching 0.702. Generally, IC_50_ values were used to reflect the antioxidant capacity of various materials. The 50% inhibitory concentration (IC_50_) was defined as the concentration of a sample that gave a 50% inhibition rate. The IC_50_ value was smaller, and the DPPH radical scavenging activity or ABTS radical scavenging activity was stronger. While the IC_50_ of DPPH radical scavenging activity of vitamin C was 0.023 mg/ml, the average IC_50_ of DPPH radical scavenging activity of CQ (0.176 mg/ml) was significantly lower than that of FQ (0.544 mg/ml) (Figure 4B). The IC_50_ of DPPH radical scavenging activity of CQ 3 was the smallest among all samples, reaching 0.100 mg/ml. While the IC_50_ of the ABTS radical scavenging activity of vitamin C was 4.437 μg/ml, the average IC_50_ of ABTS radical scavenging activity of CQ (28.98 μg/ml) was significantly lower than that of FQ (57.54 μg/ml) (Figure 4C). The IC_50_ of CQ 4 was the smallest among all samples, reaching 22.18 μg/ml. Gum et al. (2017) reported that the DPPH radical scavenging activity of 2 mg/ml RYR was approximately 20%, and that of RYR fermented with *B. subtilis* was increased to 70%. The average IC_50_ of DPPH radical scavenging activity was 0.100–0.690 mg/ml, which implied that FQ and CQ had a stronger antioxidant activity than those RYR reported previously. Huang et al. (2017) reported higher antioxidant activities in a fermented mixture of Radix Puerariae and rice than RYR due to the three times higher pigment intensity. For example, the DPPH scavenging capacity was 10.58 mg AEE/g of the sample, and the FRAP was 0.11 mmol Fe^2+^/g of the sample. Those pieces of literature were very consistent with the stronger antioxidant capacity of CQ than FQ because of the higher color value.

### Correlation Analysis Among Key Active Components and Antioxidant Activities of FQ and CQ

Correlation analysis among key active components and antioxidant activities of FQ and CQ was shown in Table 3. There was a strong correlation between the total color value (E_505_) and antioxidant activities. The correlation coefficients of FRAP, DPPH, ABTS, and E_505_ were 0.806, 0.818, and 0.822, respectively. However, there were negative correlations between FRAP and MK (*r* = −0.700), polysaccharide (*r* = −0.091), and ergosterol (*r* = −0.182). DPPH was negatively correlated with ergosterol (*r* = −0.427), polysaccharide (*r* = −0.336), MK (*r* = −0.200), and GABA (*r* = −0.046). There was a negative correlation between ABTS and MK (*r* = −0.500). Correlation analysis indicated that MonAzPs played an important role in the antioxidant activities of *Hongqu*. In contrast, the contents of MK, polysaccharide, ergosterol, and GABA were not significantly correlated with the antioxidant activities of *Hongqu*. The fermentation of the rice and Radix Puerariae mixture dramatically increased the yields of pigments and significantly improved the antioxidant capacity (Huang et al., 2017).

**TABLE 3:** Correlation analysis among key active components and antioxidant activities of FQ and CQ.

	E_50__5_	Ergosterol	MK	GABA	Polysaccharide	FRAP	DPPH	ABTS
E_5__05_	1.000	–0.184	–0.694	–0.011	–0.227	0.806**	0.818**	0.822**
Ergosterol		1.000	–0.600	0.811**	0.945**	–0.182	–0.427	0.009
MK			1.000	–0.872	–0.500	–0.700	–0.200	–0.500
GABA				1.000	0.779**	0.123	–0.046	0.241
Polysaccharide					1.000	–0.091	–0.336	0.073
FRAP						1.000	0.791**	0.918**
DPPH							1.000	0.782**
ABTS								1.000

The reciprocal of the half inhibition rate of DPPH and ABTS free radicals was used in correlation analysis. “**” indicated that significantly correlated at the 0.01 level.

### MonAzPs

**FIGURE 5:**
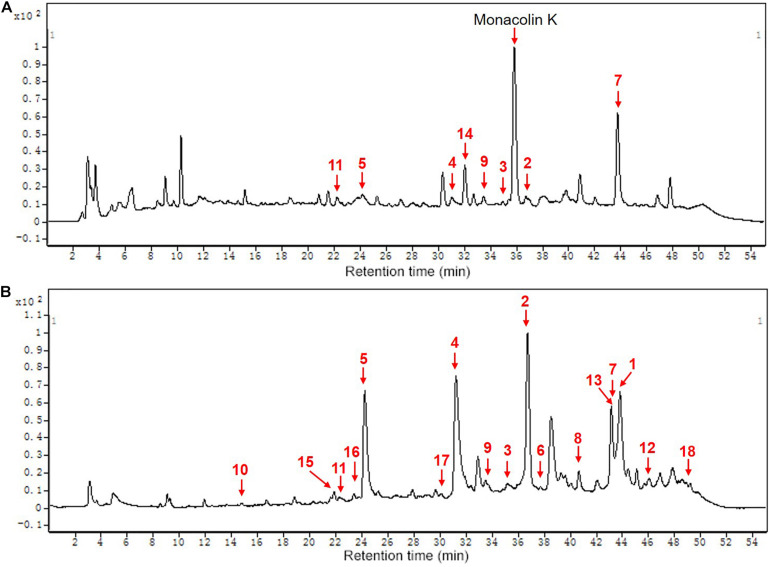
The chemical composition of MPs of functional Qu (FQ) and coloring Qu (CQ), respectively. **(A)** functional Qu (FQ). **(B)** Coloring Qu (CQ). 1, monascorubrin; 2, monascin; 3, monaphilone B; 4, monascorubramine; 5, rubropunctamine; 6, rubropunctatin; 7, ankaflavin; 8, monasfluore B; 9, monasfluore A; 10, FK17-P2B2; 11, new red pigment; 12, monapurfluore; 13, monaphilone A; 14, monascuspilion; 15, red pigment; 16, N-GABA-rubropunctatin; 17, monarubrin; 18, new yellow pigment.

HPLC-QTOF-MS was used to detect the composition of MonAzPs in FQ and CQ. As shown in Table 4 and Figure 5, a total of eighteen different MonAzPs were detected, including eleven yellow pigments (monascin, monaphilone B, ankaflavin, monasfluore B, monasfluore A, FK17-P2B2, monapurfluore, monaphilone A, monascuspilion, monarubrin, and new yellow pigment), two orange pigments (rubropunctatin and monascorubrin), and five red pigments (rubropunctamine, monascorubramine, new red pigment, N-GABA-rubropunctatin, and red pigment). It is worth noting that the chemical composition and the relative abundance of yellow pigments in FQ and CQ were quite different. There were only eight different MonAzPs (five yellow pigments and three red pigments) in FQ (Figure 5A), and the relative abundance of yellow pigments (monascuspilion, monasfluore A, monaphilone B, and monascin) was higher than that of the others. There were seventeen different MonAzPs (eleven yellow pigments, four red pigments, and two orange pigments) in CQ (Figure 5B), and the relative abundance of yellow pigments (monascin, ankaflavin, and monaphilone A) was greater than that of other MonAzPs. The differences of MonAzPs may be the reason for their different antioxidant activities.

**TABLE 4:** *Monascus* pigments composition.

No.	Tr/min	Identification	Color	Max absorbance	Formula	Proposal ions	Experimental m/z	References
1	43.81	Monascorubrin	Orange	472	C_23_H_26_O_5_	[M + H]^+^	383.1846	Manchand et al., 1973
2	36.68	Monascin	Yellow	386	C_21_H_26_O_5_	[M + H]^+^	359.1865	Chen et al., 1969; Salomon and Karrer, 1932
3	35.39	Monaphilone B	Yellow	390	C_20_H_28_O_4_	[M + H]^+^	333.2049	Hsu et al., 2010
4	31.54	Monascorubramine	Red	529	C_23_H_27_NO_4_	[M + H]^+^	382.2000	Birch et al., 1962; Sweeny et al., 2012
5	24.19	Rubropunctamine	Red	528	C_21_H_23_NO_4_	[M + H]^+^	354.1702	Birch et al., 1962; Sweeny et al., 2012
6	37.01	Rubropunctatin	Orange	473	C_21_H_22_O_5_	[M + H]^+^	355.1538	Chen et al., 1969
7	43.18	Ankaflavin	Yellow	390	C_23_H_30_O_5_	[M + H]^+^	387.2168	Manchand et al., 1973
8	40.65	Monasfluore B	Yellow	390	C_23_H_28_O_5_	[M + H]^+^	385.2017	Huang et al., 2008
9	33.47	Monasfluore A	Yellow	386	C_21_H_24_O_5_	[M + H]^+^	357.1690	Huang et al., 2008
10	14.77	FK17-P2B2	Yellow	390	C_13_H_16_O_4_	[M + H]^+^	237.1114	Jongrungruangchok et al., 2004
11	22.59	New red pigment	Red	520	C_23_H_27_NO_5_	[M + H]^+^	398.1951	Liang et al., 2019
12	45.67	Monapurfluore	Yellow	390	C_23_H_32_O_4_	[M + H]^+^	373.2372	Hsu et al., 2010
13	43.17	Monaphilone A	Yellow	390	C_22_H_32_O_4_	[M + H]^+^	361.2372	Hsu et al., 2010
14	32.04	Monascuspilion	Yellow	390	C_21_H_28_O_5_	[M + H]^+^	361.2009	Chiu et al., 2012
15	22.19	Red pigment	Red	520	C_21_H_26_O_6_	[M + H]^+^	375.1797	Liang et al., 2019
16	23.38	N-GABA-rubropunctatin	Red	520	C_25_H_29_NO_6_	[M + H]^+^	440.2072	Chen et al., 2017
17	30.15	Monarubrin	Yellow	390	C_20_H_26_O_4_	[M + H]^+^	331.1901	Loret and Morel, 2010
18	49.44	New yellow pigment	Yellow	410	C_23_H_28_O_6_	[M + H]^+^	401.2349	Chen et al., 2017

**FIGURE 6:**
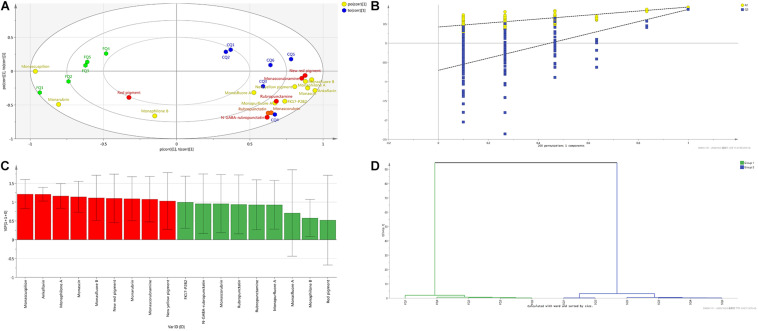
Orthogonal projections to latent structures-discriminant analysis (OPLS-DA) of *Monascus* pigments composition of functional Qu (FQ) and coloring Qu (CQ), respectively. **(A)** Bioplot diagram of FQ (green hexagon), CQ (blue hexagon), and *Monascus* pigments (red, yellow, orange dots). **(B)** Permutation test diagram of OPLS-DA model (*R*^2^ = 0.421, *Q*^2^ = –0.706). **(C)** VIP diagram. **(D)** HCA diagram.

From the bioplot diagram (Figure 6A) and the HCA diagram (Figure 6D), FQ (green hexagon) and CQ (blue hexagon) were located on different sides of the y-axis or divided into different clusters, which indicates that FQ and CQ had significant differences. Red, orange, and yellow dots were used to represent red, orange, and yellow pigments, respectively. The smaller distance between *Hongqu* and MPs indicated that *Hongqu* played a more important role in MPs. CQ and most of the MPs were located on the right side of the y-axis, indicating that most of the MPs came from CQ. The distance between FQ and monascuspilion was smaller than that between CQ and monascuspilion, which meant monascuspilion mainly came from FQ. This was consistent with the results in Figure 5. According to the permutation test diagram of the OPLS-DA model (Figure 6B), it can be seen that the OPLS-DA model was effective and not overfit (*R*^2^ > 0.4). According to the VIP diagram (Figure 6C), there were nine different MPs with a significant difference between FQ and CQ (Supplementary Table 1, VIP > 1), including monascuspilion, ankaflavin, monaphilone A, monascin, new red pigment, monarubrin, monascorubramine, and new yellow pigment.

### Antioxidant Activity of MYPs

#### Antioxidant Activity of MYPs *in vitro*

**FIGURE 7:**
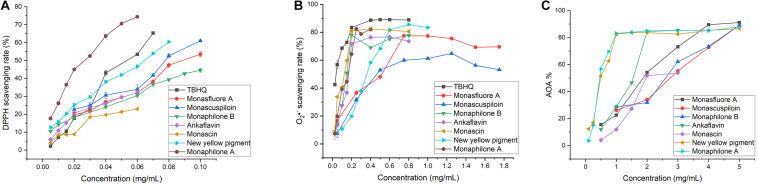
*In vitro* antioxidant of yellow pigments purified from functional Qu (FQ) and coloring Qu (CQ). **(A)** The DPPH radical scavenging activity of yellow pigment from FQ and CQ. **(B)** The superoxide anion radical scavenging activity of yellow pigment from FQ and CQ. **(C)** The inhibition rate of yolk lipoprotein peroxidation of yellow pigment from FQ and CQ.

The antioxidant activities of seven MYPs were evaluated by antioxidant assay *in vitro*, including the DPPH free radical scavenging rate (Figure 7A), superoxide anion clearance rate (Figure 7B), and inhibition rate of peroxidation of yolk lipoprotein (Figure 7C). The three chemical assays reveal the different antioxidant characteristics of MYPs based on different antioxidant mechanisms. The antioxidant capacity of MYPs increased with the increase of concentration. The antioxidant activities of MYP were similar in the three chemical antioxidant assays. Chemical antioxidant tests revealed that the antioxidant activities of monaphilone A, ankaflavin, and new yellow pigment only separated from CQ were significantly stronger than monasfluore A and monascuspilion only separated from FQ. For example, the IC_50_ of the new yellow pigment in three chemical assays (0.062, 0.376, and 0.580 mg/ml) was smaller than that of monascuspiloin (0.080, 0.548, and 2.670 mg/ml). This might be the main reason for the significantly stronger antioxidant capacity of the crude ethanol extract of CQ than FQ. MYPs have good DPPH radical scavenging ability and superoxide anion scavenging capacity, and bad vitellin inhibitory capacity. The IC_50_ of the DPPH free radical scavenging rate of MYPs from FQ (0.080–0.091 mg/ml) was smaller than the IC_50_ of crude ethanol extracts of FQ (0.544 mg/ml). The results indicated that the antioxidant activity of yellow pigments was improved significantly after purification.

#### Cellular Antioxidant Activity of MYPs

**FIGURE 8:**
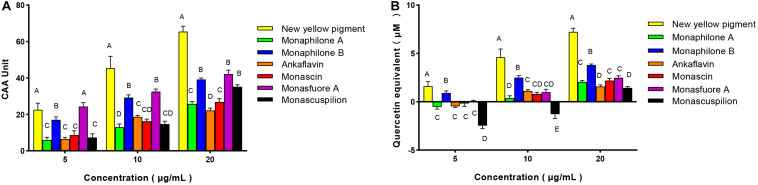
Cellular antioxidant activity (CAA) of *Monascus* yellow pigment from functional Qu (FQ) and coloring Qu (CQ) and quercetin equivalent in Caco-2 cells. **(A)** Cellular antioxidant activity value. **(B)** Quercetin equivalent.

Compared with the antioxidant assay *in vitro* and animal experiments, the evaluation of antioxidant activity by cell model test was more economical and faster. It is widely used to quantify the cellular antioxidant activity (CAA) of phytochemicals, food extracts, and nutritional supplements. Therefore, we consider CAA assays as reliable and sensitive methods for antioxidative activity evaluation. With quercetin as a positive control, CAA of seven pigments was evaluated with an oxidative damage model of human colon adenocarcinoma Caco-2 cells induced by AAPH (Kellett et al., 2018). The CAA value of MYPs at the 20 μg/ml concentration showed that the new yellow pigment (65.46 units) had the best antioxidant activity, followed by monasfluore A (42.15 units) and monaphilone B (39.19 units), which were all significantly better than monascin (26.86 units) and ankaflavin (22.19 units), the two most frequently reported MYPs (Figure 8A). The antioxidant capacity of the natural product was usually converted to an equivalent concentration of quercetin based on its CAA values for making the result more comparable (Zhou et al., 2020). The quercetin equivalent of MYPs was shown in Figure 8B. The quercetin equivalent of the new yellow pigment was 7.23 μM. In short, the new yellow pigment had outstanding antioxidant capacity *in vitro*, and its antioxidant capacity *in vivo* was worthy of in-depth study.

**FIGURE 9:**
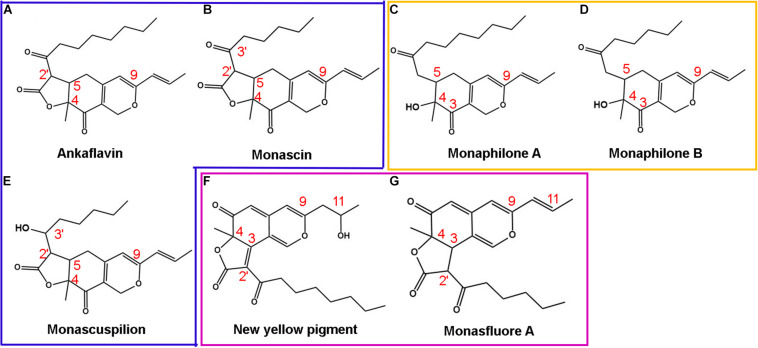
Structures of *Monascus* yellow pigments. **(A)** ankaflavin, **(B)** monascin, **(C)** monaphilone A, **(D)** monaphilone B, **(E)** monascuspilion, **(F)** new yellow pigment, and **(G)** monasfluore A. Similar structures are represented by different colored boxes. *Monascus* yellow pigments (MYPs) in the blue box have a C5-C2′ linear tricyclic carbon skeleton, MYPs in the orange box do not have a lactone ring, and MYPs in the purple box have a C3-C2′ angular tricyclic carbon skeleton.

With the help of *in vitro* and *in vivo* antioxidant experiments, the relationship between the structure and antioxidant activity was further investigated. Based on the structure of 111 identified single pigment, the tricyclic carbon skeleton structure or the dicyclic carbon skeleton was the core structure of MonAzPs, which generally contained one to three carbonyl groups and two to five unsaturated double bonds. To date, a unitary trunk pathway had been revealed to produce four classical pigments and intermediates. All other 106 species MonAzPs were generated by various shunt pathways branching off from the trunk pathway (Chen et al., 2019). First, the alterations in the position and number of hydroxyl groups affect the antioxidant activity. The hydroxyl group of C-4 was prone to condensation reaction to form a lactone ring C5-C2′ condensation and cyclization formed a C5-C2′ linear tricyclic carbon skeleton or C3-C2′ condensation and cyclization formed a C3-C2′ angle tricyclic carbon skeleton. The seven yellow pigments could be divided into three categories according to carbon skeleton characteristics and the CAA. New yellow pigment and monasfluore A (Figures 9F,G) had an angular tricyclic carbon skeleton, obviously different from the linear tricyclic core of classical monascin, ankaflavin, and monascuspilion (Figures 9A,B,E). It is worth noting that the antioxidant activity of the new yellow pigment and monasfluore A was stronger and that of monascin, ankaflavin, and monascuspilion. Monaphilone A and monaphilone B had moderate antioxidant activity lacking the γ-lactone ring (instead of a C-4 hydroxyl group) (Figures 9C,D). First of all, alterations of the hydroxyl group of MYPs affect the antioxidant activities significantly. The results showed that the new yellow pigment with a hydroxyl group on C-11 possessed much higher antioxidant activities than monasfluore A and the others with a C10 (11) double bond on C-11. Monascuspilion with a hydroxyl group on C-3′ brought higher antioxidant activities than monascin with a ketone group on C-3′. In the case of MYPs, alterations of the γ-lactone ring and alkyl side chain could affect the antioxidant activities slightly. Monaphilone A and monaphilone B (lacking γ-lactone ring) possessed higher antioxidant activity than ankaflavin and monascin (linear tricyclic core), respectively. Moreover, monaphilone B and monascin (*R* = C_5_H_11_) had higher antioxidant activities than monaphilone A and ankaflavin (*R* = C_7_H_15_). In short, the alterations that occurred at C-3′, C-11, and the carbon skeleton more obviously affected the antioxidant activities.

The inhibitory effects of azaphilonoid pigments on 12-O-tetradecanoylphorbol-13-acetate (TPA)-induced ear edema were much stronger than those of non-azaphilonoid pigments (Akihisa et al., 2005). The critical structures that enhance the anti-inflammatory activity of *Monascus* yellow and orange pigments were found by Hsu (Hsu L. C. et al., 2013), and the structure–activity relationship was consistent with the results of the relationship between the structure and antioxidant activity of MYPs in this article. Because inflammation is a kind of exogenous stress, the Keap1-Nrf2/ARE antioxidant signal pathway is often activated (Lee B. H. et al., 2013). Nrf2 is an important transcription factor that regulates cell protection mechanisms such as antioxidant and anti-inflammatory activities (Pall and Levine, 2015). Therefore, anti-inflammatory activity is closely related to antioxidant activity. It is worth noting that MonAzPs with hydroxyl groups on C-3′, C-11 had significantly stronger antioxidant activity than a ketone group and C10 (11) double bond on C-3′, C-11, respectively. The previous studies had reported that the antioxidant potential of MYPs could easily transfer hydrogen to and accept electrons from free radicals through HAT and SET mechanisms. This result was consistent with the alterations of key antioxidant structures of MYPs.

## Conclusion

This study analyzes the difference of key active components from two types of *Hongqu* (FQ and CQ). It found that MonAzPs may play an essential role in the antioxidant activity. The antioxidant activities of monaphilone A, ankaflavin, and new yellow pigment only from CQ are significantly more potent than monasfluore A and monascuspilion only from FQ. The new yellow pigment has the best antioxidant activity, whereas monascin and ankaflavin, the two most frequently reported MYPs, have the worst antioxidant activity. MYPs with hydroxyl groups on C-3′ and C-11 are proved to displace higher antioxidant activity than MYPs with a ketone group or C10 (11) double bond on C-3′ and C-11. Thus, it provides a potential antioxidant resource for functional foods. Future studies should focus on the antioxidant activities *in vivo* and the potential mechanism to better elucidate the antioxidant mechanisms *via* hydrogen atom transfer and single electron transfer.

## Data Availability Statement

The raw data supporting the conclusions of this article will be made available by the authors, without undue reservation.

## Author Contributions

LW performed the research and wrote the manuscript. KZ and FC participated in collecting raw materials and analyzing the metabolites. GC and YY calculated and analyzed the data. XL, WZ, PR, and LN designed the study. All authors reviewed and approved the final version of the manuscript.

## Conflict of Interest

The authors declare that the research was conducted in the absence of any commercial or financial relationships that could be construed as a potential conflict of interest.

## Publisher’s Note

All claims expressed in this article are solely those of the authors and do not necessarily represent those of their affiliated organizations, or those of the publisher, the editors and the reviewers. Any product that may be evaluated in this article, or claim that may be made by its manufacturer, is not guaranteed or endorsed by the publisher.
