# Supplementary material for: Comparative Study on the Antioxidant Activity of Monascus Yellow Pigments From Two Different Types of Hongqu—Functional Qu and Coloring Qu
Source: Front Microbiol. 2021 Aug 2;12:715295. doi: 10.3389/fmicb.2021.715295 (PMC8365423; doi:10.3389/fmicb.2021.715295)
Supplement: Supplementary file 1 [file Data_Sheet_1.doc]

Supplementary Material

# Supplementary Tables

## Supplementary Table 1 | Monascus pigments significantly associated based on VIP value

| Monascus pigments | VIP | Coefficient |
| --- | --- | --- |
| Monascuspilion | 1.212 | 0.387 |
| Ankaflavin | 1.210 | 0.181 |
| Monaphilone A | 1.163 | 0.329 |
| Monascin | 1.142 | 0.412 |
| Monasfluore B | 1.112 | 0.602 |
| New red pigment | 1.101 | 0.643 |
| Monarubrin | 1.091 | 0.585 |
| Monascorubramine | 1.077 | 0.605 |
| New yellow pigment | 1.030 | 0.754 |

Note: VIP, variable importance in projection. VIP>1 .

## Supplementary Table 2 | The half inhibition rate and linear equation of anti-oxidation in vitro of monascus yellow pigments

|  | DPPH IC_50_ /(mg/mL) | DPPH linear equation | O_2_^•-^ IC_50_ /(mg/mL) | O2•^-^ linear equation | PUFA IC_50_ /(mg/mL) | PUFA linear equation |
| --- | --- | --- | --- | --- | --- | --- |
| TBHQ | 0.048 | y = 1199.0x - 7.062, R^2^=0.985 | 0.024 | y = 194.5x + 45.27,R² = 0.962 | 0.060 | y = 903.8x + 2.376,R² = 0.996 |
| New yellow pigment | 0.062 | y = 621.2x + 11.28,R² = 0.991 | 0.376 | y = 148.7x - 5.956,R² = 0.988 | 0.580 | y = 81.08x + 3.076,R² = 0.970 |
| Monaphilone A | 0.030 | y = 1080.x + 17.71,R² = 0.951 | 0.167 | y = 314.3x + 2.397,R² = 0.981 | 0.520 | y = 92.30x - 2.402,R² = 0.944 |
| Monasfluore A | 0.091 | y = 449.4x + 8.987,R^2^=0.988 | 0.452 | y = 82.62x + 12.65,R² = 0.972 | 2.070 | y = 17.24x + 14.35,R² = 0.912 |
| Monascuspiloin | 0.080 | y = 498.7x + 10.21,R^2^=0.970 | 0.548 | y = 75.11x + 8.856,R² = 0.932 | 2.670 | y = 16.46x + 6.028,R² = 0.985 |
| Monaphilone B | 0.113 | y = 347.5x + 10.86,R^2^=0.989 | 0.140 | y = 380.6x - 3.260,R² = 0.970 | 2.570 | y = 16.26x + 8.248,R² = 0.955 |
| Ankaflavin | 0.123 | y =285.1x + 14.95,R^2^=0.996 | 0.205 | y = 225.8x + 3.708,R² = 0.993 | 1.400 | y = 47.06x - 15.85,R² = 0.955 |
| Monascin | 0.235 | y = 154.8x + 13.64, R² = 0.995 | 0.117 | y = 349.7x + 9.182,R² = 0.938 | 2.080 | y = 31.68x - 15.86,R² = 0.948 |

Note: TBHQ, tert-butyl hydroquinone.

# Supplementary Figures

**
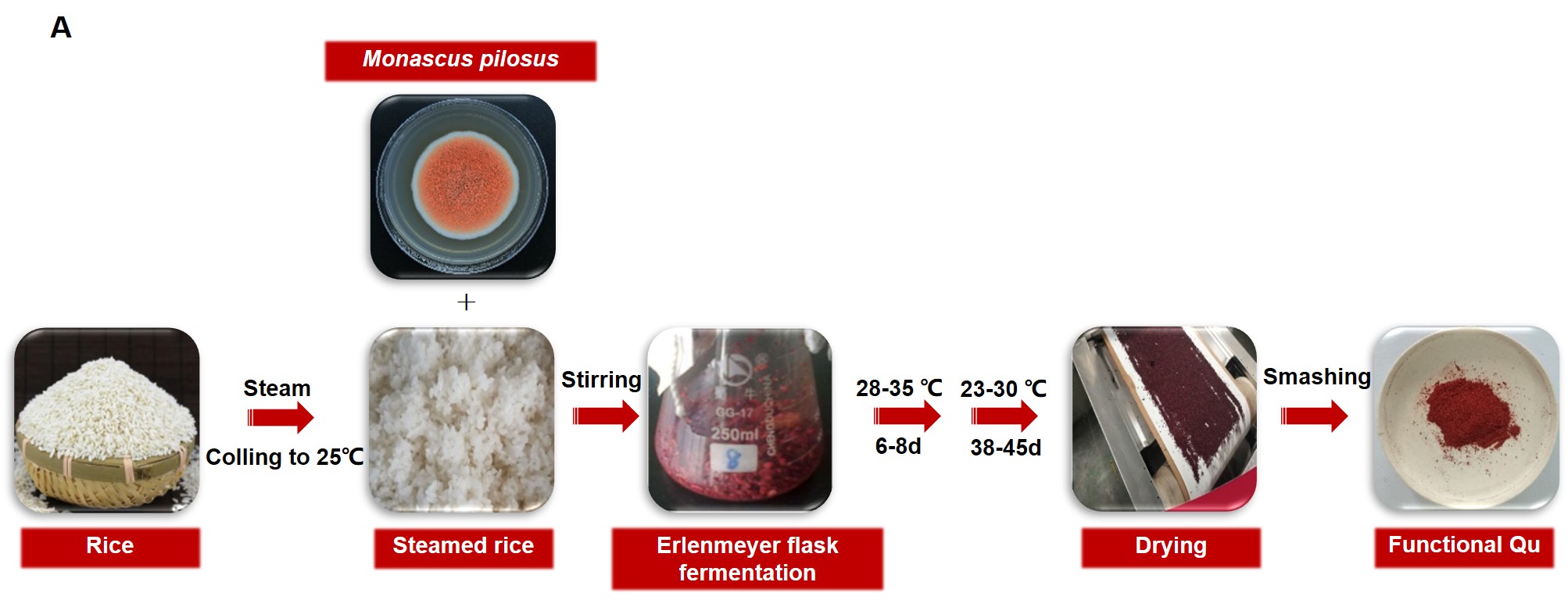
**

**
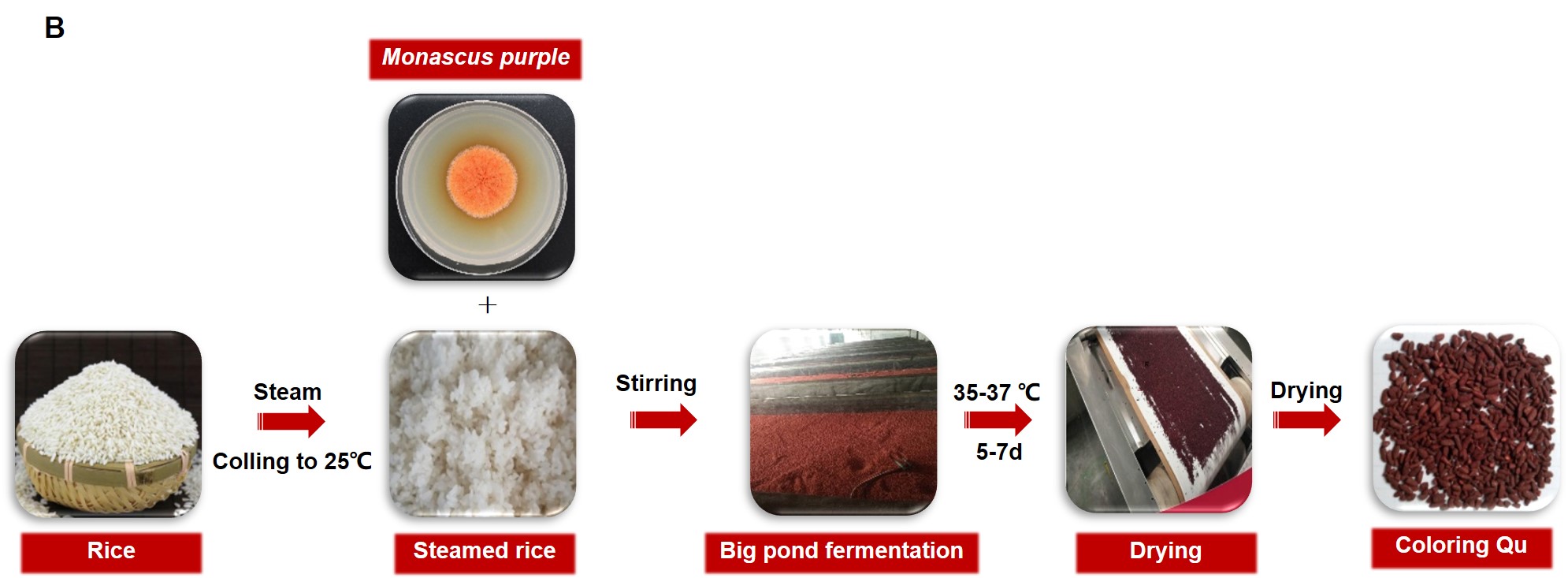
**

**Supplementary Figure 1.** The traditional processing technologies of (A) functional Qu and (B) coloring Qu in China.

**
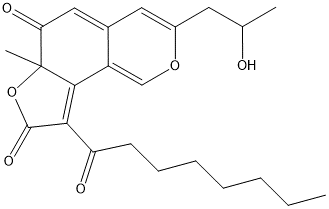
**

**Supplementary Figure 2.** The high performance liquid chromatogram, mass spectrum, and molecular structure of new yellow pigment (C_23_H_28_O_6_).


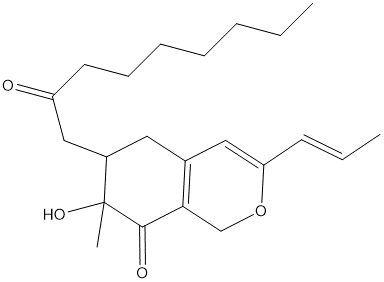


**Supplementary Figure 3.** The molecular structure, high performance liquid chromatogram, mass spectrum of monaphilone A (C_22_H_32_O_4_).


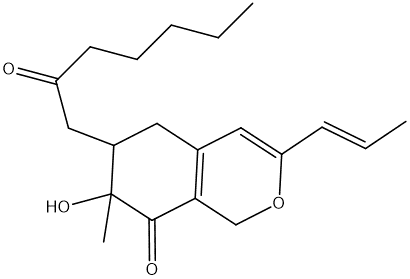


**Supplementary Figure 4.** The molecular structure, high performance liquid chromatogram, mass spectrum of monaphilone B (C_20_H_28_O_4_).


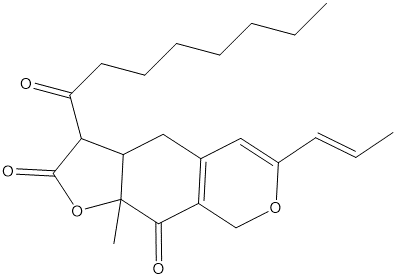


**Supplementary Figure 5.** The molecular structure, high performance liquid chromatogram, mass spectrum of ankaflavin (C_23_H_30_O_5_).


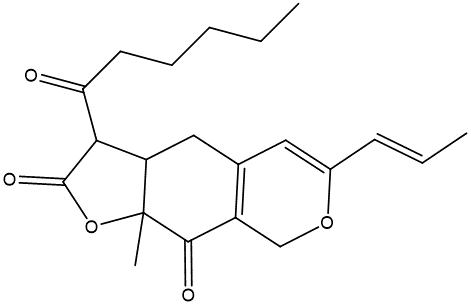


**Supplementary Figure 6.** The molecular structure, high performance liquid chromatogram, mass spectrum of monascin (C_21_H_26_O_5_).


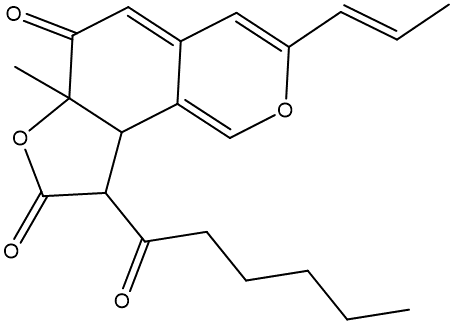


**Supplementary Figure 7.** The molecular structure, high performance liquid chromatogram, mass spectrum of monasfluore A (C_21_H_24_O_5_).


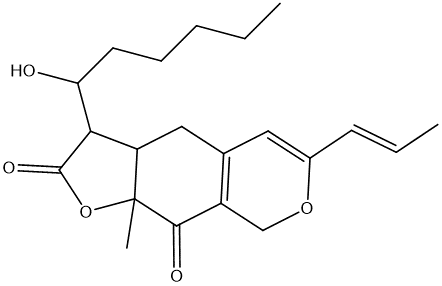


**Supplementary Figure 8.** The molecular structure, high performance liquid chromatogram, mass spectrum of monascuspilion (C_21_H_28_O_5_).
